# The Story of the Insane from Year to Year

**Published:** 1896-10-03

**Authors:** 


### THE STORY OF THE INSANE FROM YEAR TO YEAR.

Ninth Series.

### EDINBURGH ROYAL ASYLUM.

Here the numbers were practically stationary during the year, being 882 on January 1st, and 884 on December 31st. There were 336 first admissions, 74 readmissions, 170 recoveries, 122 discharged relieved, and 96 deaths. The percentage of recoveries calculated on the admissions stands at 41·5, and the deaths at 10·9 per cent. on the average number resident. Of the deaths 51 were caused by cerebral and spinal diseases, 10 by consumption, 4 by enteritis, and 1 by small-pox. Intemperance in drink is given as the cause of insanity in 87 of the year's admissions, hereditary influence in 134, religion in 5, and old age in 55. The new annexe, known as Craig House, although formally opened in 1894, was not occupied by patients until 1895. It is well known to be one of the most advanced buildings in the world for the treatment of the insane, and Dr. Clouston expresses his satisfaction at having almost ideal conditions at command for the treatment of his patients; and he further remarks that the treatment of mental disease depends largely on a series of complicated conditions. This is so, but undoubtedly a well-planned asylum is a great help to the medical man, and it is very questionable whether a really well-planned asylum exists south of the Tweed. The asylum contains about 360 private patients; the rest being paupers. Private patients are received at rates varying from £28 to £1,000 a year. In addition to the medical superintendent there are five medical assistants, so that the institution is thoroughly well officered—over-manned, one would almost say.

### WORCESTER COUNTY ASYLUM.

There was an increase of 21 patients during 1895, and at the end of the year 1,038 patients were in residence. There were 181 first admissions, 20 readmissions, 64 recoveries, 7 discharged relieved, and 76 deaths. The recoveries stand at 34 per cent. of the admissions, and the deaths at 7·4 of the average number resident. Thirty-two of the deaths were caused by cerebral and spinal diseases, 13 by consumption, 2 by pneumonia, and 1 to typhoid fever. Hereditary tendency existed in 26 of the admissions, and intemperance in drink in 18. When speaking of the classification of patients, Dr. Cooke says that if expense were no object this classification might be improved by multiplying the wards and reducing the number of patients in each, allowing a larger area per head than is now required by the regulations. But such a scheme would involve much expense both in building and working, and Dr. Cooke thinks the number of cures would not be increased. We fully agree with with Dr. Cooke in this; and it is surely reasonable in everything connected with rate-supported institutions that the element of expense should be considered. At the present day the initial cost of asylums strikes us as being altogether excessive. We hear £150 a bed, and sometimes £200, mentioned as the sums which such-and-such an asylum. It is always pleasing to note the granting of pensions to asylum officials, and we are glad to learn that David Wagstaff obtained £41 18s. 9d. a year after thirty-four years' service. The asylum is about to be enlarged to 1,200 beds—and the visitors very wisely remark that it is not desirable to go beyond this. We would ask, is it desirable to go so far? The visitors congratulate themselves upon the retention in their service of Dr. Cooke. The weekly cost per head is 7s. 9⅝d.

### HULL BOROUGH ASYLUM.

There were 402 patients in residence at the close of the year, the number having increased from 387. There were 120 first admissions; 27 readmissions; 47 recoveries; 28 discharged relieved, and 51 deaths. The recoveries stand at 31·97 per cent on the admissions, and the deaths at 12·97 on the average number resident. Of the deaths 33 were caused by cerebral and spinal diseases; 7 by consumption, and 1 by enteritis. Intemperance in drink accounts for 34 of the year's admissions; hereditary influence for 42, and domestic trouble for 6. The number of patients increases by about fifteen every year, and the additions now in course of construction will only suffice for eight years, when another invasion of bricks and mortar will take place; and so it will go on until the Hull people realise what drink and heredity really mean. Fifty per cent. of last year's admissions were due to these causes alone! In the meantime, Dr. Merson advises the erection of a detached block for private patients; and he truly adds that such a house would be more than self-supporting, and would prove a boon to people who are desirous of maintaining their relatives as private patients. Mr. Mackintosh, the head attendant, obtained a pension of £52 a year, after twenty years' service. We congratulate Mr.

Mackintosh, but we regret to learn that ill-health was the cause of resignation. The weekly cost per head was 11s. 1d.

MIDDLESEX COUNTY ASYLUM AT WANDSWORTH.

On January 1st this asylum contained 1,121 patients, and by December 31st the number had risen to 1,179. There were 346 first admissions, 49 readmissions, 137 recoveries, 39 relieved, and 84 deaths during the year. The recoveries were 37 per cent. calculated on the admissions, and the deaths were 7·2 on the average number resident. Thirty-seven of the deaths were due to cerebral and spinal diseases, and 10 to consumption. Domestic trouble was the cause of insanity in 35 of the admissions, intemperence in drink in 32, and hereditary influence in 47. The asylum is full, and 98 patients are boarded out in other asylums; but an annexe for idiot children is being built, which will relieve the pressure to some extent. The relief thus obtained will be both slight and temporary, and something else must be done to meet the constant claims of the insane. To add further to an asylum which will contain 1,400 beds would be utterly foolish, and it is to be hoped the committee will act upon Dr. Hill's advice, and at once set about providing another asylum. Two of the subordinate staff received pensions during the year, and Dr. Hill well says that these pensions have had "a beneficial effect upon the asylum." The committee "record their high appreciation of the able services rendered by the medical superintendent." The weekly cost of maintenance is 10s. 7d. per head.

